# Coronary Artery Fistula Aneurysm: Pathological Analysis After Surgery

**DOI:** 10.7759/cureus.14903

**Published:** 2021-05-08

**Authors:** Atsuo Mori, Shinya Inoue, Hideki Orikasa, Tatsuji Yoshimoto, Hiroaki Konishi

**Affiliations:** 1 Cardiovascular Surgery, Kawasaki Municipal Hospital, Kawasaki, JPN; 2 Cardiovascular Surgery, Kawasaki Municipal Kawasaki Hospital, Kawasaki, JPN; 3 Pathology, Kawasaki Municipal Kawasaki Hospital, Kawasaki, JPN; 4 Cardiology, Kawasaki Municipal Ida Hospital, Kawasaki, JPN

**Keywords:** coronary artery fistula aneurysm, coronary artery fistula, cabg, segmental arterial mediolysis, median arcuate ligament syndrome, abdominal visceral aneurysm

## Abstract

An asymptomatic 75-year-old woman was identified with a 40-mm-sized, round-shaped lesion beside the pulmonary artery on computed tomography (CT). Coronary angiography showed a coronary artery fistula (CAF) with an aneurysm branching from the left anterior descending artery toward the pulmonary artery. The CAF aneurysm (CAFA) was resected and coronary artery bypass graft surgery using the left internal thoracic artery was performed successfully. Pathological analysis revealed that medial depletion similar to segmental arterial mediolysis (SAM) may contribute to aneurysm formation.

## Introduction

Coronary artery fistula (CAF) is a rare coronary anomaly accounting for 0.3% of congenital heart diseases [1-2]. CAF is defined as an abnormal coronary artery termination into any cardiac chamber or great vessel, bypassing the myocardial capillary network. The prevalence of CAF seen on computed tomography (CT) angiography is reported to be as high as 0.9%, which is higher than the previously reported prevalence of 0.002%-0.3% at cardiac angiography [3-4].

CAF may, on rare occasions, be accompanied by the formation of an aneurysm as a CAF aneurysm (CAFA). CAFA with a small diameter is often asymptomatic and requires no surgical intervention. Although it is extremely rare that the diameter exceeds 30 mm, there have been reports that the surgical resection of a large CAFA is necessary because it can cause cardiac tamponade and sudden death due to rupture [5]. However, due to its rarity, as far as we know, there have been few detailed pathologic examinations or images of CAFA. Here, we encountered a case of large CAFA and used the full-resected specimen for pathological examination, including special collagen staining and immunostaining. Furthermore, we pointed out that the histological image was similar to that of an abdominal visceral aneurysm and discussed it, including its clinical implications.

## Case presentation

A 75-year-old woman was referred to our outpatient clinic for deteriorated renal function with proteinuria. She had already been treated for type 2 diabetes mellitus and hypertension. She had no complaints, including chest pain or dyspnea on exertion. No heart murmurs were identified on auscultation.

**Figure 1:**
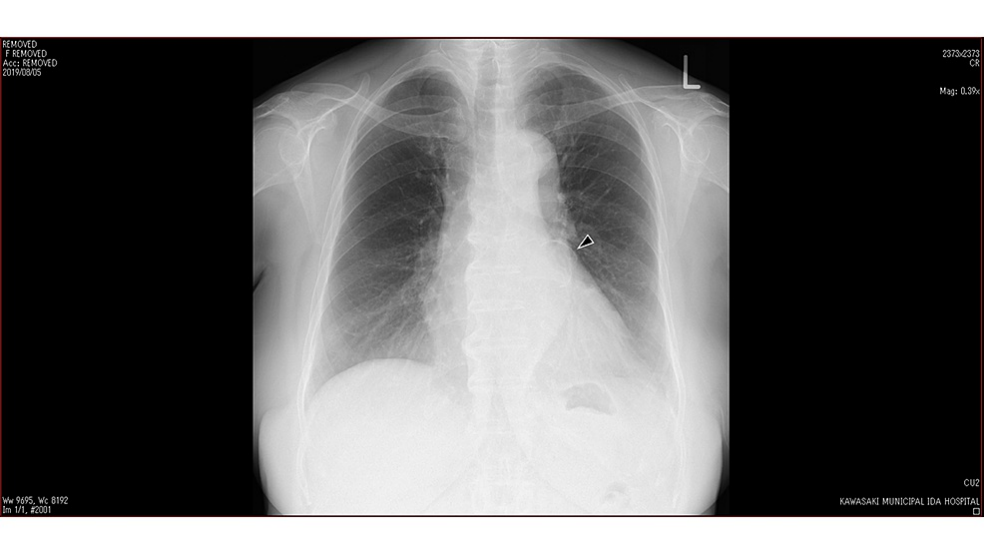
Chest X-ray Chest X-ray shows a round, hypoechoic lesion (black arrow) with calcification.

Blood testing revealed: blood urea nitrogen (BUN) 39.6mg/dl; creatinine 2.0 mg/dl; and estimated glomerular filtration rate (eGFR) 17.2 mL/min/1.73 m^2^. Glycated hemoglobin (HbA1c) was 6.8%, urinary protein was 3+, and urinary sugar was negative. Brain natriuretic peptide (BNP) value was 18.9 pg/ml. Electrocardiography (ECG) was normal. Chest X-ray revealed an enlarged heart, and a round, calcified lesion was identified next to the pulmonary artery (PA) (Figure 1).

**Figure 2:**
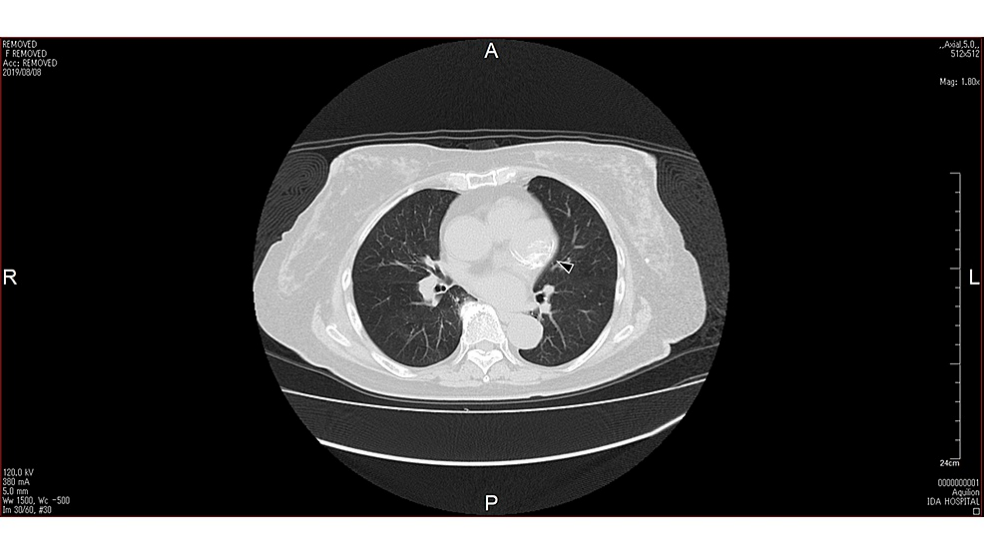
Computed tomography Computed tomography shows a spherical mass (black arrow) with 40 mm of calcification in the cardiac sac outside the heart and next to the PA. PA: pulmonary artery

Computed tomography (CT) without contrast enhancement was performed with the intention of ruling out malignancy of the kidney. By chance, a spherical mass with 40 mm of calcification was identified in the cardiac sac outside the heart and next to the PA (Figure 2).

**Figure 3:**
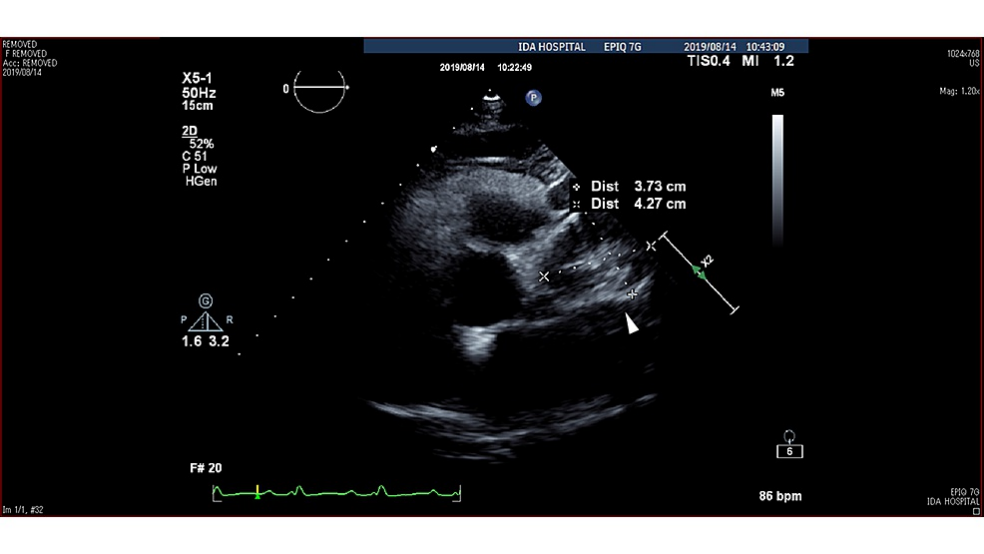
Transthoracic echocardiography Transthoracic echocardiography shows an elliptical hypoechoic lesion (white arrow) with a diameter of 43x37 mm between the PA and left coronary artery. A thrombus-like region of moderate echogenicity is present inside the lesion. PA: pulmonary artery

Transthoracic echocardiography (TEE) also demonstrated an elliptical hypoechoic lesion measuring 43x37 mm between the PA and left coronary artery (LCA) (Figure 3).

The shunt into the PA could not be visualized using Doppler. Left ventricular function was within normal range, and the ejection fraction was 66%. Cine-mode magnetic resonance imaging (MRI) revealed a well-defined, 4 cm mass on the cranial side of the left ventricle and on the left side of the main PA. The mass was in contact with the LCA, and blood flow and thrombi consistent with pulsation were apparent in the mass (Video 1).

**Video 1:** Cine-mode MRI Cine-mode MRI shows a well-defined, 4 cm mass (white arrows) on the cranial side of the left ventricle and on the left side of the main PA. Blood flow and thrombus consistent with pulsation are found in the mass. PA: pulmonary artery

Cardiac catheterization revealed a blood vessel that appeared to represent fistula from the left anterior descending artery (LAD) to the PA. An enlarged blood vessel that appeared to be the lumen of the aneurysm was evident along the way. The left main trunk was also dilated to 8 mm in diameter (Video 2).

**Video 2:** CAG CAG shows a large CAFA arising from the LAD and then connecting towards the PA. The left main trunk is enlarged. The LAD and LCX show neither stenosis nor dilatation. CAG: coronary angiogram; CAFA: coronary artery fistula aneurysm; PA: pulmonary artery; LAD: left anterior descending artery; LCX: left circumflex artery

Both the LAD and left circumflex artery (LCX) displayed neither stenosis nor dilatation. The right coronary artery was shown to remain intact.

The results of oxygen saturation sampling were: right atrium (RA) 69.0%; right ventricle (RV) 66.5%; PA main 70.1%; PA right 68.5%; PA left 69.3%; and pulmonary artery wedge (PAW) 95.5%. Pressure studies showed: RA -1 torr; RV (systolic/end-diastolic/diastolic), 28/-3/0 torr; PA (systolic/diastolic/mean), 21/1/12 torr; and mean PAW pressure, 0 torr. The pulmonary to systemic flow ratio was 1.05.

At the time of myocardial stress scintigraphy, heart rate suddenly decreased to 30 beats/min immediately after adenosine triphosphate loading, and the examination had to be interrupted. No ischemic findings were seen from resting myocardial scintigraphy. Although we fully understood the utility of three-dimensional (3D)-CT, we avoided 3D-CT using contrast media in consideration of the patient’s renal dysfunction.

**Figure 4:**
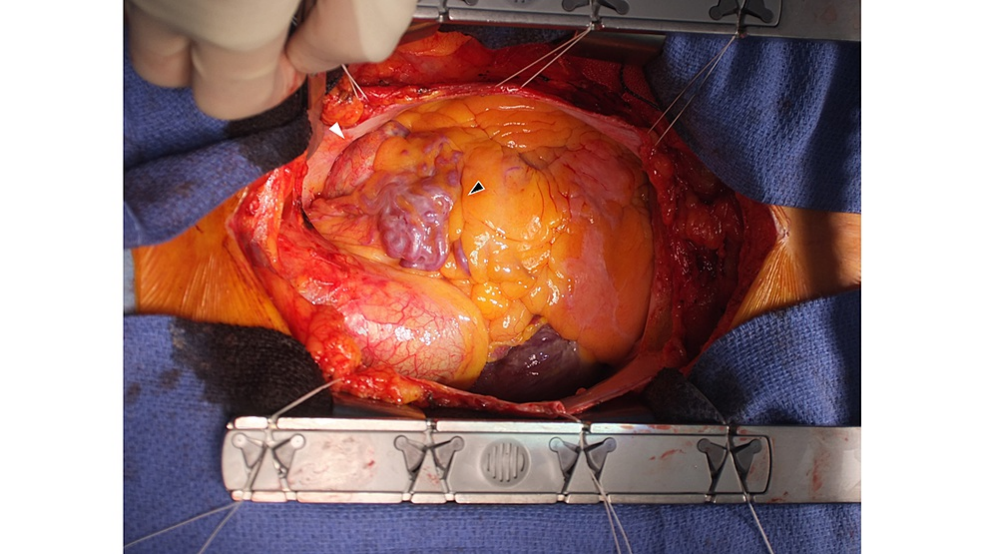
Intraoperative view Intraoperative surgeon’s view. Varicose vein-like blood vessels are found in the right ventricular outflow tract (black arrow). A calcified, round mass is next to the PA (white arrow). PA: pulmonary artery

Considering the size of the aneurysm (diameter >30 mm), we recommended aneurysmal resection by open heart surgery to prevent rupture. The patient consented to undergo surgery, so surgery was performed. When the pericardium was incised after median sternotomy, varicose vein-like blood vessels were first found in the right ventricular outflow tract (Figure 4).

**Figure 5:**
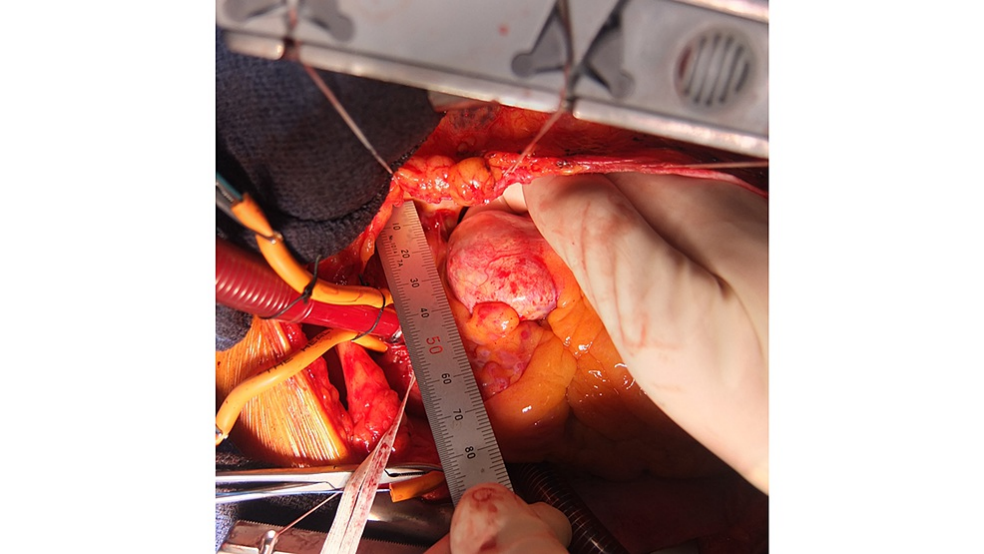
Intraoperative inspection Intraoperative inspection. A calcified, egg-shaped mass is found between the base of the PA and LCA. PA: pulmonary artery; LCA: left coronary artery

Next, when the heart was abducted to the right, a round mass was seen between the base of the PA and the LCA, continuous with the vine-shaped blood vessels. Appearance and palpation were similar to an aneurysm with calcification (Figure 5).

In other words, an aneurysm had formed in the middle of a fistula from the LCA to the PA, as diagnosed preoperatively.

**Figure 6:**
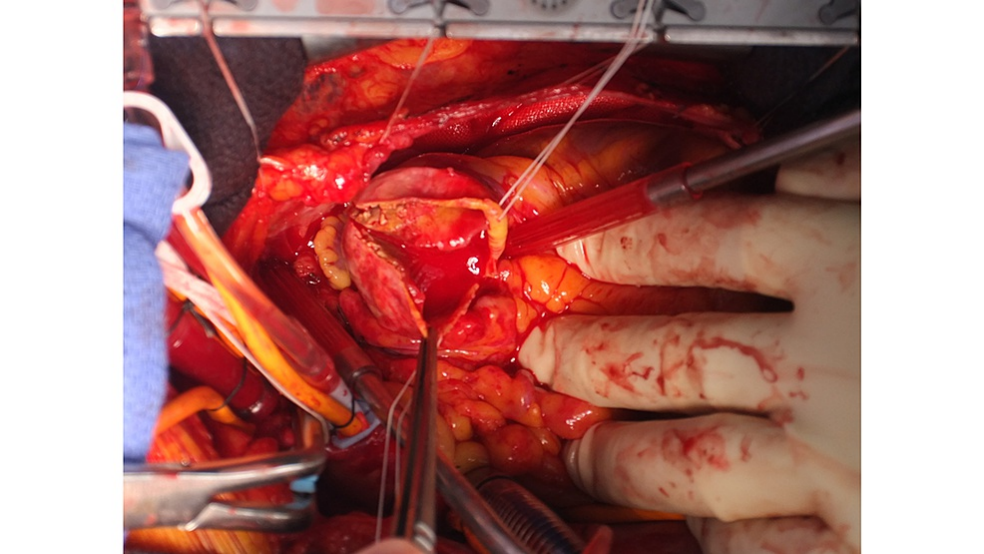
CAFA with an incision The CAFA is incised under cardiac arrest strong arteriosclerosis with mural thrombus inside. CAFA: coronary artery fistula aneurysm

A heart-lung machine was established and a cardioplegic solution (CP) was injected to obtain cardiac arrest. Considering CAF may cause the steal of an antegrade cardioplegic solution, we added a retrograde CP injection, which induced complete cardiac arrest. Full resection of the CAFA was intended. A mural thrombus was found in a small incision of the aneurysm. From inside the CAFA, branch vessels other than inflow and outflow could not be identified (Figure 6).

**Figure 7:**
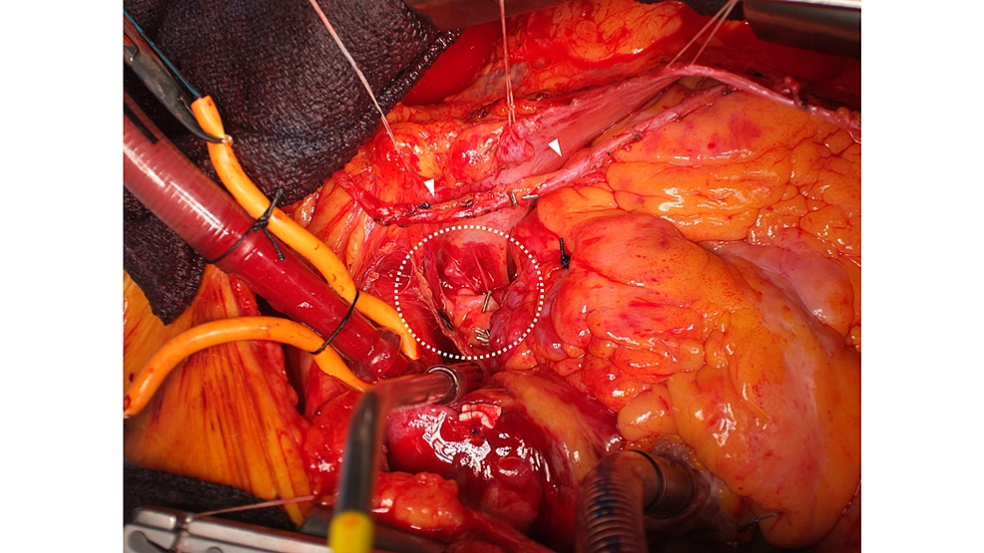
CABG after resection of CAFA CABG of LITA (white arrow) to the LAD is performed. The CAFA (dot circle) is resected fully. CAFA: coronary artery fistula aneurysm; LITA: left internal thoracic artery

We ligated the fistula vessels from the LAD. In addition, the vascular branch from the anterior descending branch to the CAF was particularly fragile and could readily cause bleeding. Finally, the LAD was ligated at both the central and peripheral sites of this branch. We then performed bypass surgery of the left internal thoracic artery (LITA) to the LAD. Thereafter, we resected the CAFA fully (Figure 7).

Vine-like vessels in the right ventricular outflow tract were ligated with surgical clips as much as possible but not all were resected due to bleeding. Withdrawal from the heart-lung machine was smooth and the postoperative course was uneventful. The patient was discharged and has remained healthy without cardiovascular events for 10 months thereafter.

**Figure 8:**
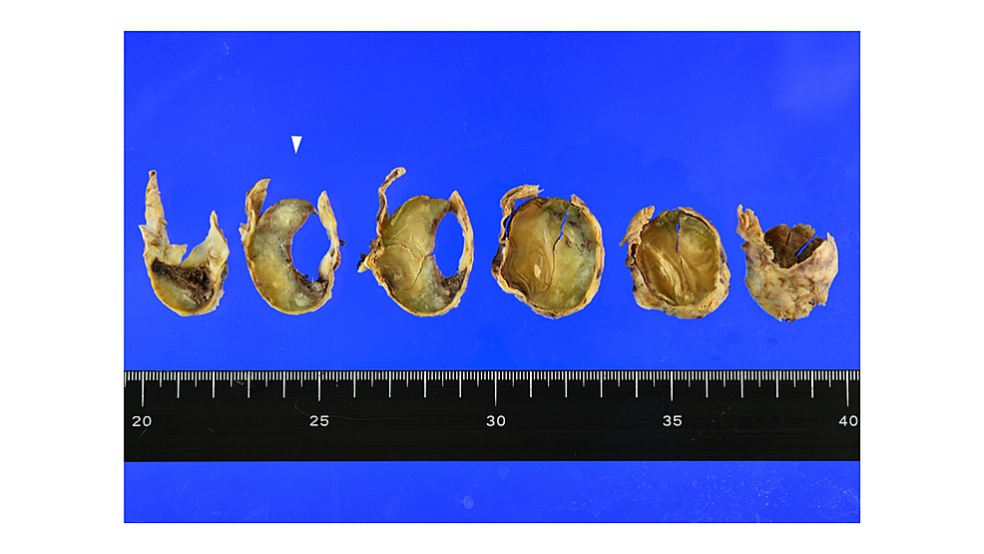
Macroscopic findings Macroscopic findings. Cross-sections of resected CAFA show uneven thickness of the arterial wall, accompanied by calcification. The inside of the aneurysm is filled with thrombus. The second slice from the left (white arrow) is explored using light microscopy. CAFA: coronary artery fistula aneurysm

The macroscopic findings corresponded to a saccular aneurysm. When viewed in cross-section, the thickness of the arterial wall was uneven and accompanied by calcification. The inside of the CAFA was found to be filled with atherosclerotic thrombus (Figure 8).

**Figure 9:**
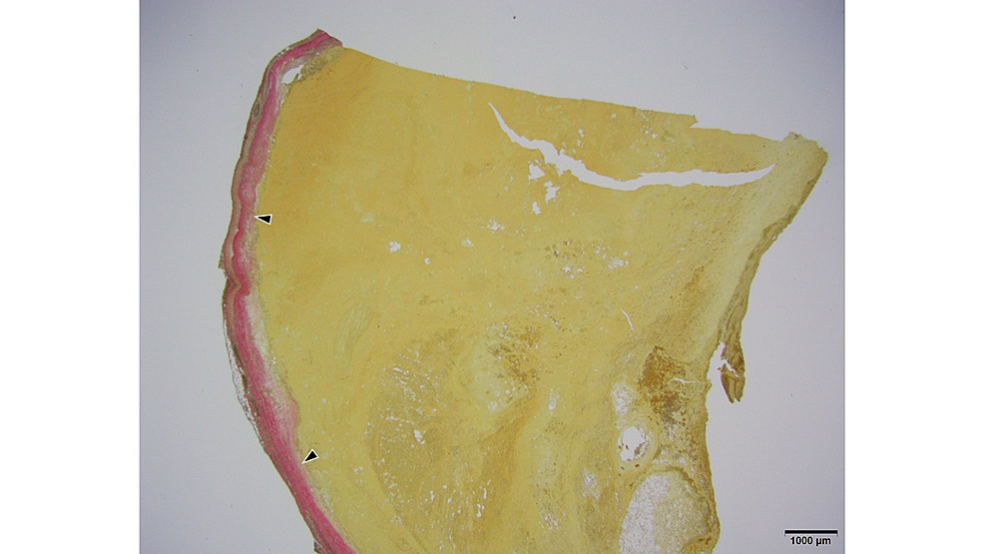
Microscopic finding with EVG Thinnest aneurysm wall where the media is extensively defective (black arrows). The inside of the CAFA is filled with atherosclerotic thrombus. EVG: Elastica Van Gieson; CAFA: coronary artery fistula aneurysm

**Figure 10:**
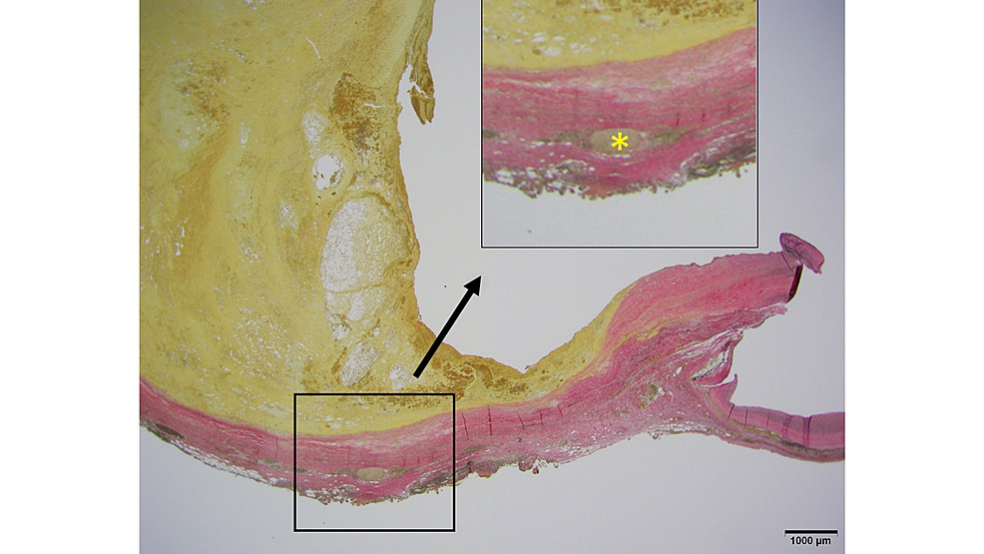
Microscopic finding with EVG Most parts of the aneurysm consist of adventitia and intima. The media is found (asterisk) segmentally. EVG: Elastica Van Gieson

**Figure 11:**
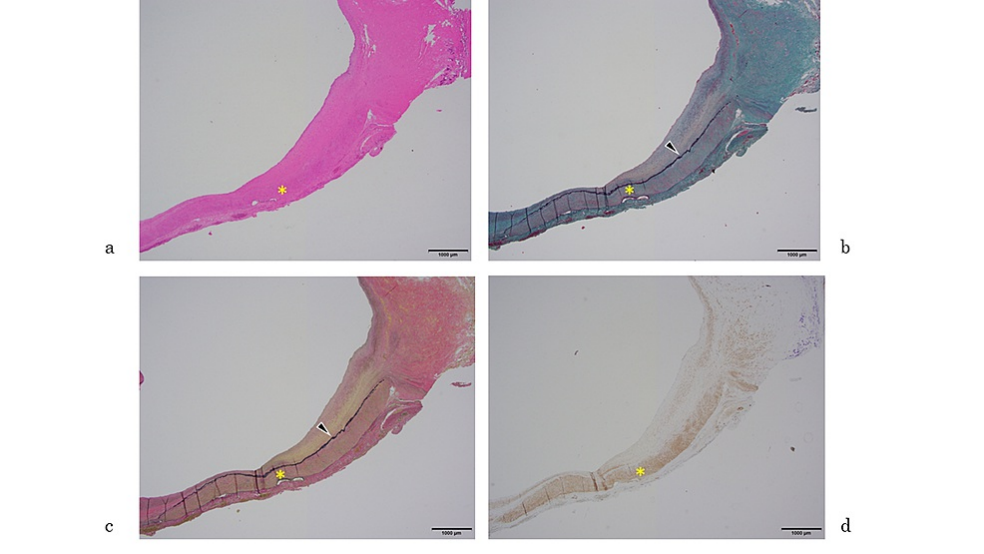
Microscopic findings a. Hematoxylin and eosin (HE) staining; b. Elastica Masson (EM) staining; c. Elastica Van Gieson (EVG) staining; d. Smooth muscle actin (SMA) immunostaining Images using HE and various other stains. Media (asterisk) and internal elastic plate (black arrow) are also confirmed by immunostaining with SMA.

**Figure 12:**
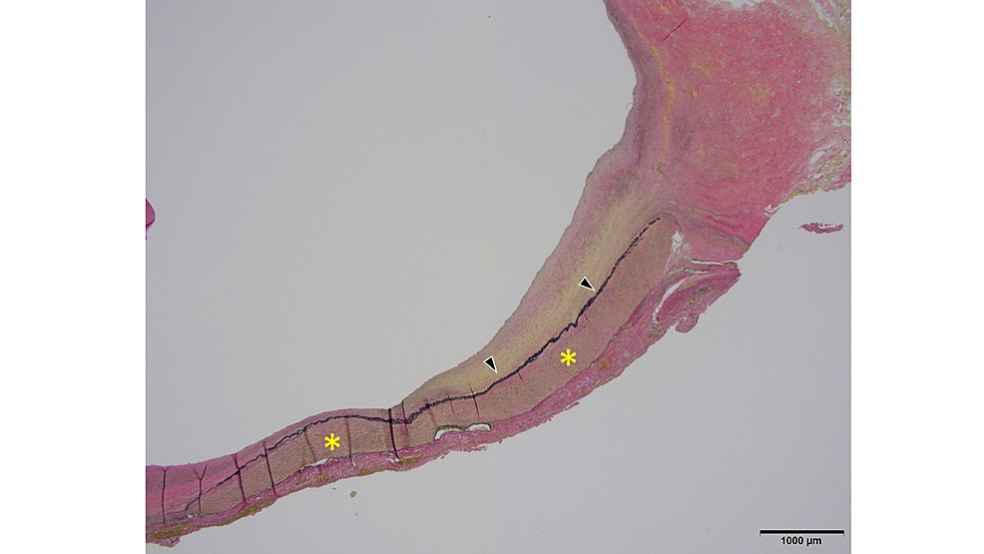
Microscopic finding with EVG The media (asterisks) and inner elastic plate (black arrows) are stained with EVG, showing interruptions. EVG: Elastica Van Gieson

Microscopically, an aneurysm showing atherosclerosis was recognized. Most of the aneurysms comprised adventitia and intima, and the media was extensively defective and only partially evident (Figures 9-10). The formation of atheroma was widely observed in the intima, and the inner and outer elastic plates had disappeared extensively. The medial loss was also confirmed by immunostaining for smooth muscle actin (SMA) (Figures 9-12).

No thickening of the intima or adventitia or strong inflammatory findings, as findings characteristic of coronary aneurysms due to Kawasaki disease, were observed.

## Discussion

CAF is primarily a congenital coronary malformation, with a reported frequency of about 0.8%. The most frequent origin of CAF is the right coronary artery (50%), followed by the LAD (35%) and LCX. The PA is the most frequent end point (89%), followed by the right ventricle, right atrium, left atrium, and coronary sinus [1]. In most cases, patients remain asymptomatic and often require no special treatment. However, large amounts of shunt may cause chest pain and heart failure, necessitating surgical intervention.

CAF is rarely accompanied by the formation of CAFA. When the aneurysm is small, the risk of rupture is considered low. However, if the CAFA is large, a risk of rupture has been reported, leading to cardiac tamponade and sudden death [5]. According to past reports, CAFA tends to be more common among elderly people in their 70s and women, and many reports have described cases in Asians, especially from Japan [6].

CAFA is thought to be caused by the addition of arteriosclerosis to congenital CAF. An aberrant vessel that grows as a kind of collateral channel has few elastic fibers in the blood vessel wall. When excessive stress is applied to the wall due to increased blood flow or inflammation, elastic fibers are often destroyed and aneurysm formation may tend to result. Pathological examination of the fully resected CAFA has been sparse due to the rarity of this entity. The resected specimen showed strong atherosclerosis. Special staining revealed characteristic findings such as a defect in the media and the internal elastic plate. A well-known coronary aneurysm is associated with Kawasaki disease, which is an acquired disease that often occurs with high fever in children.

The pathological images in this case clearly differed from those of coronary aneurysms associated with Kawasaki disease [7]. Specifically, the fibrosis and thickening of both the intima and adventitia in a coronary aneurysm associated with Kawasaki disease were not observed in this case. Pathological images of CAFA and Kawasaki disease aneurysms should be investigated to facilitate the differentiation of these entities.

The pathological images in this case were similar to the pathological findings of an abdominal visceral aneurysm. Segmental arterial mediolysis (SAM) is a disease of unknown etiology that mainly causes mediolysis of the muscular arteries in abdominal organs [8]. Median arcuate ligament compression syndrome (MALS) results from chronic stenosis of the origin of the celiac artery due to extrinsic compression and presents as epigastric ischemia [9]. Both pathologies are known to form aneurysms in the abdominal cavity that rupture, causing intra-abdominal hemorrhage.

Similar to MALS, the mismatch between blood flow stress and vascular wall strength might cause CAFA. Progression of arteriosclerosis and loss of the media may be the most important factors in the formation and enlargement of CAFA.

In the diagnosis of CAFA, a comprehensive evaluation by combining diagnostic imaging modalities, including CAG and TTE, is important. The recent literature has reported that image information from contrast-enhanced 3D-CT is particularly useful [10]. In cases with renal dysfunction, cine-mode MRI, which does not use a contrast medium, might be useful for differential diagnosis.

At present, three options are available for the treatment of CAFA. The first is a strict observation of the clinical course. The prognosis of small, asymptomatic CAFA is considered generally favorable and rupture is rare. As a result, small, asymptomatic CAFA can be followed under proper follow-up using imaging modalities. The second option is surgical treatment. The surgical application for asymptomatic CAFA remains controversial. However, recent reports have described large CAFA rupturing, leading to death due to cardiac tamponade or shock [11].

Lowe et al. described surgical management of CAFA as appropriate in symptomatic patients who display evidence of emboli from aneurysm to the distal coronary bed leading to myocardial ischemia and in cases of aneurysmal enlargement (diameter >30 mm) as documented by serial angiographic measurement [12].

According to our case, CAFA is also pathologically extremely vulnerable in the absence of media. At this point, we think that the surgical indications for CAFA are basically in line with the indications for abdominal visceral aneurysm showing similar histopathological features.

Past literature suggests that aggressive intervention is recommended because of the high risk of rupture if the size of the aneurysm exceeds 30 mm [13-14]. However, the rupture of an aneurysm is not determined only by the diameter, and cases of rupture have been reported when the aneurysm shape is irregular even if the diameter is smaller than 10 mm [15]. Surgical resection may also be preferred in cases of small CAFA less than 30 mm in diameter tending to expand rapidly.

Catheter intervention, including coil embolization of CAF, is the third option. The problem with this intervention is that delivering the catheter distal to a severely tortuous CAF can be difficult. Complete occlusion of both the inflow and outflow channels of CAFA can also be often difficult. Although experience with using the option is sparse at present, it might be justified for the treatment of elderly or high-risk patients.

The number of cases of large CAFA may increase with patient age and advances in diagnostic tools. We believe that the accumulation of experience will lead to the establishment of appropriate therapeutic algorithms for this pathology.

## Conclusions

Pathological analysis revealed that medial depletion similar to segmental arterial mediolysis (SAM) may contribute to aneurysm formation. Surgical resection may be preferred in cases of small CAFA that are less than 30 mm in diameter and tend to expand rapidly.
